# Feasibility and Perceived Impact of an AI-Assisted Pre-Matriculation Study Strategies Curriculum for Incoming Medical Students

**DOI:** 10.7759/cureus.111895

**Published:** 2026-07-01

**Authors:** Claire Pishko, Troy J Weinstein, Jacqueline Bergen, Katie Kowalek, Daniel C Butler, Elaine Situ-LaCasse, Richard Amini

**Affiliations:** 1 Medicine, University of Arizona College of Medicine, Tucson, USA; 2 Pediatrics, University of Arizona College of Medicine, Tucson, USA; 3 Dermatology, University of Arizona College of Medicine, Tucson, USA; 4 Emergency Medicine, University of Arizona College of Medicine, Tucson, USA

**Keywords:** artificial intelligence, evidence-based learning, large language models, learning science, medical education, study strategies

## Abstract

Background

Many incoming medical students begin training using study approaches that were effective in prior academic settings but may not meet the pace and cognitive demands of medical school. Evidence-based strategies such as retrieval practice and spaced repetition improve long-term retention, yet students are rarely formally taught how to apply them. Large language models (LLMs) may offer a scalable way to develop accessible educational materials that introduce these concepts to students before matriculation.

Objective

The objective of this study was to evaluate the feasibility, engagement, and perceived impact of an eight-week, AI-assisted "study strategies" curriculum for incoming medical students.

Methods

All incoming students at a single medical school received weekly emails summarizing evidence-based study techniques, with supplementary PDF summaries and podcasts. Content was drafted using LLMs and reviewed for accuracy. Students completed a voluntary survey assessing demographics, prior instruction on study strategies, engagement with materials, and perceived impact. Descriptive statistics summarized outcomes. Group comparisons used t-tests and ANOVA, and Pearson correlations assessed relationships between engagement and perceived impact.

Results

A total of 65% (78/120) students completed the survey. Students read a mean of 3.58 emails (SD=2.53), reviewed 2.29 PDFs (SD=2.22), and listened to 1.03 podcasts (SD=1.76). Participants reported learning new techniques they planned to use (M=2.09, SD=0.63), moderate influence on study approach (M=2.58, SD=0.85), and moderate likelihood of applying strategies in medical school (M=3.10, SD=1.14). Greater email engagement correlated with perceived helpfulness, learning new techniques, influence on study approach, and likelihood of application (r=0.302-0.612, all p<0.01). No significant differences were observed by first-generation status, prior formal instruction, or path to medical school.

Conclusion

This AI-assisted pre-matriculation study strategies curriculum was feasible to implement and was perceived as useful by participating students. Higher engagement with curricular materials was associated with greater perceived impact on study approaches and planned application of evidence-based learning strategies.

## Introduction

Medical students enter training with diverse academic experiences and study habits, yet many rely on approaches that served them well previously but are not always sufficient for the volume and complexity of medical training. Evidence from cognitive psychology demonstrates that techniques such as spaced repetition, retrieval practice, interleaving, elaboration, and dual coding lead to stronger learning and improved long-term retention compared with standard study methods [1]. Despite this, explicit instruction in evidence-based learning strategies is inconsistently provided in undergraduate or medical curricula, leaving many students unaware of the practices most likely to support their success.

Within medical education, structured instruction in effective learning strategies has been associated with improved examination performance, deeper understanding of foundational science, and enhanced retention of complex material [2]. Nevertheless, students frequently report uncertainty about how to study efficiently for medical school and often rely on familiar patterns from prior coursework [3]. Providing guidance on evidence-based strategies before the start of the academic year may help students transition more effectively into medical training, particularly those from diverse educational backgrounds or without prior exposure to the learning sciences.

Pre-matriculation offers an especially advantageous moment for intervention. During this period, students are motivated, receptive, and not yet burdened by the demands of formal coursework, creating an opportunity to introduce techniques that can shape study habits from the outset. However, developing high-quality, engaging materials that translate learning-science evidence into practical guidance can be time-consuming for educators.

Large language models (LLMs) may offer a scalable approach to translating evidence from cognitive psychology into practical educational resources. Although effective study strategies are well established in the learning sciences, many medical schools may lack faculty with specific expertise in this area or the time required to develop comprehensive instructional materials. LLMs can assist educators by rapidly generating summaries, examples, and learner-centered content based on educational literature, potentially reducing the burden of curriculum development. However, AI-generated content should not be considered inherently accurate or educationally effective. Rather, LLMs may serve as a tool to support the creation of instructional materials that are subsequently reviewed, refined, and aligned with educational evidence by faculty. This approach may allow institutions to disseminate evidence-based study strategies more efficiently while maintaining educational quality and oversight.

This study describes the development and evaluation of an eight-week pre-matriculation curriculum for incoming medical students that introduced evidence-based study strategies through weekly emails, PDF summaries, and brief podcasts. We hypothesized that the curriculum would be feasible to implement, that students would engage with the materials, and that greater engagement would be associated with higher perceived usefulness and anticipated application of the strategies during medical school.

## Materials and methods

This study was cross-sectional and conducted at the University of Arizona College of Medicine in Tucson, Arizona. The study was reviewed by the University of Arizona Institutional Review Board (Submission ID: STUDY00006876) and approved as a Quality Improvement (QI) project that did not constitute human subjects research.

Emails were sent to all 120 accepted medical students matriculating in July 2025 at the University of Arizona College of Medicine-Tucson. The intervention period was May 25, 2025, through July 21, 2025, during which eight weekly "Study Saturdays" emails were distributed. Participation in reviewing the emails, PDFs, and podcasts was voluntary. Feasibility was assessed through successful delivery of the curriculum, student participation in the intervention and survey, engagement with curricular materials, and implementation without major technical barriers. The survey was administered during July orientation. Survey participation was anonymous, voluntary, and had no impact on students' academic standing.

The intervention consisted of an eight-week pre-matriculation curriculum introducing evidence-based learning strategies relevant to medical education. Weekly emails provided an overview of a specific study technique, summarized key principles from the learning sciences, and offered practical guidance for implementation. To accommodate different learning preferences, each email also included an extended two-page PDF summary and a brief audio podcast of approximately 10-15 minutes elaborating on the week's topic. A curriculum map outlining the weekly topics, learning objectives, and educational materials is provided in Table 1.

**Table 1:** Curriculum map of the eight-week pre-matriculation study strategies curriculum

Week	Topic	Learning objective	Study strategy covered
1	How to learn effectively in medical school	Describe evidence-based approaches to learning and identify common ineffective study habits.	Foundations of effective learning; introduction to learning science principles
2	Spaced repetition	Explain the benefits of spaced repetition and apply spacing principles to long-term retention.	Spaced repetition
3	Six evidence-based learning strategies	Compare multiple evidence-based study techniques and identify appropriate applications for each.	Retrieval practice, spaced repetition, elaboration, dual coding, concrete examples, and interleaving
4	How to mix related content	Explain how alternating related topics can improve learning and transfer of knowledge.	Interleaving
5	Retrieval-based learning strategies	Apply active recall techniques to strengthen memory and knowledge retention.	Retrieval practice
6	Answering challenging multiple-choice questions	Develop strategies for approaching complex examination questions and identifying knowledge gaps.	Test-enhanced learning and retrieval-based practice
7	Fostering metacognition and learning	Evaluate personal learning strategies and monitor understanding during the learning process.	Metacognition and self-monitoring
8	Optimizing self-regulated study	Develop a structured and intentional approach to planning, monitoring, and adapting study behaviors.	Self-regulated learning

Curriculum materials were developed using a combination of peer-reviewed educational literature, educator oversight, and large language model (LLM) tools. For each weekly topic, relevant literature addressing evidence-based learning strategies was identified and reviewed by an educator with experience in medical education and academic support. Key concepts and practical applications were synthesized into an educational framework tailored to incoming medical students. ChatGPT (OpenAI, San Francisco, CA, USA) was used to assist in drafting learner-facing content, including weekly email summaries and PDF materials. LLM-generated content served as an initial draft and was subsequently reviewed and revised to ensure consistency with the source literature, clarity of communication, and appropriateness for the target audience. Podcast materials were generated using NotebookLM (Google Labs, Mountain View, CA, USA) after relevant source materials were uploaded into the platform. All materials were reviewed, edited, and approved by the senior author prior to dissemination to ensure accuracy, educational relevance, and alignment with the underlying literature. Development, review, editing, and distribution of the weekly materials required approximately four hours per week, including literature review, content preparation, quality review, and dissemination. Students were not specifically informed that LLMs were used in the development of the curricular materials.

Students completed a survey (Appendix 1) at orientation that assessed demographic characteristics, engagement with the study strategies materials, and perceived impact of the curriculum. Demographic items included first-generation medical student status, prior formal instruction in study strategies, and path to medical school (direct entry from undergraduate studies, one to two gap years, more than two gap years, or career changer). Engagement was assessed by asking students to report the number of emails, PDF summaries, and podcasts they engaged with on a scale from zero to eight. Objective engagement metrics, such as email open rates, PDF downloads, and podcast analytics, were not available. Perceived impact was measured using four items: helpfulness of the email series, whether the series introduced new study techniques students planned to use, the extent to which the series influenced their approach to studying, and the likelihood that they would apply the strategies during medical school. The items used Likert-type scales appropriate to each construct.

Descriptive statistics were used to summarize demographic characteristics, engagement, and perceived impact. Independent sample t-tests were conducted to compare perceived-impact measures by first-generation status and prior formal instruction. Differences across paths to medical school were assessed using one-way analysis of variance. Pearson correlation coefficients were calculated to examine associations among engagement and perceived-impact variables. Parametric methods were selected because Likert-type outcome measures were treated as approximately continuous variables, consistent with common practice in educational research. These analyses were intended to explore relationships among engagement and perceived-impact measures rather than establish causal effects. Statistical significance was defined as p < 0.05. No formal correction for multiple comparisons was applied, and subgroup and correlational analyses should be interpreted as exploratory.

## Results

A total of 78 out of 120 incoming medical students completed the survey, indicating a response rate of 65%. Of these, 58 students (74.4%) identified as first-generation medical students and 20 (25.6%) did not. Forty-six students (59.0%) reported prior formal instruction in study strategies, while 32 (41.0%) reported no such instruction. Regarding the path to medical school, 22 students (28.2%) matriculated directly from undergraduate studies, 33 (42.3%) reported one to two gap years, 19 (24.4%) reported more than two gap years, and four (5.1%) identified as career changers. These demographics are represented in Table 2.

**Table 2:** Participant demographics of the 2025 entering class (n = 78)

Characteristic	n (%)
Total respondents	78 (65 %)
First-generation medical student	58 (74.4 %)
Prior study strategy instruction	46 (59.0 %)
Path to medical school	
Direct from undergraduate studies	22 (28.2 %)
1-2 gap years	33 (42.3 %)
> 2 gap years	19 (24.4 %)
Career changer	4 (5.1 %)

Engagement with the study strategies curriculum varied across participants. Students reported reading an average of 3.58 of the eight emails (SD = 2.53), reviewing 2.29 PDF summaries (SD = 2.22), and listening to 1.03 podcasts (SD = 1.76). This can be seen in Table 3.

**Table 3:** Engagement with the "study strategies" content

Engagement measure	Mean (SD)
Emails read (0-8)	3.58 (2.53)
PDFs reviewed (0-8)	2.29 (2.22)
Podcasts listened (0-8)	1.03 (1.76)

Perceived impact, shown in Table 4, of the curriculum was modest. Participants indicated that the series somewhat introduced them to new study techniques they planned to use (M = 2.09, SD = 0.63; coded 1 = No, 2 = Somewhat, 3 = Yes). The extent to which the series influenced or changed their approach to studying was rated between slight and moderate (M = 2.58, SD = 0.85; coded 1 = Not at all, 5 = Extremely). Students also reported that they were moderately likely to apply the strategies discussed in the series during their medical school studies (M = 3.10, SD = 1.14; coded 1 = Not at all likely, 5 = Extremely likely).

**Table 4:** Perceived impact of the study strategies curriculum

Question	Mean (SD)	Scale
Helpfulness of the email series (1-5)	2.95 (0.95)	1 = Not at all helpful; 5 = Extremely helpful
Likelihood of recommending the series to future students (1-3)	3.07 (1.03)	1 = No; 2 = Somewhat; 3 = Yes
Introduced new study techniques (1-5)	2.09 (0.63)	1 = Not at all; 5 = Extremely
Influence on approach to studying (1-5)	2.58 (0.85)	1 = Not at all; 5 = Extremely
Likelihood of applying strategies (1-5)	3.10 (1.14)	1 = Not at all likely; 5 = Extremely likely

Subgroup analyses (Table 5) revealed no significant differences in perceived helpfulness, learning new techniques, influence on study approach, or likelihood of applying strategies based on first-generation status (all p > 0.05), prior instruction in study strategies (all p > 0.05), or path to medical school (all p > 0.05).

**Table 5:** Subgroup analyses of perceived impact

Subgroup comparison	Group means (SD)	p-value
Q8: Helpfulness		
First-generation vs non-first-generation	3.00 (0.86) vs 2.90 (1.17)	0.684
Prior formal study strategy instruction vs none	3.12 (1.10) vs 2.87 (0.81)	0.24
Path to medical school	Direct: 3.13 (1.10); 1-2 gap years: 2.94 (0.92); >2 gap years: 2.86 (0.85); career changer: 2.50 (1.00)	0.342
Q10: New Techniques		
First-generation vs non-first-generation	2.02 (0.61) vs 2.30 (0.66)	0.082
Prior formal study strategy instruction vs none	2.19 (0.69) vs 2.02 (0.58)	0.245
Path to medical school	Direct: 2.27 (0.55); 1-2 gap years: 2.09 (0.72); >2 gap years: 1.95 (0.52); career changer: 1.75 (0.50)	0.281
Q12: Influence on Study Approach		
First-generation vs non-first-generation	2.55 (0.86) vs 2.65 (0.81)	0.657
Prior formal study strategy instruction vs none	2.66 (0.90) vs 2.52 (0.81)	0.493
Path to medical school	Direct: 2.73 (0.83); 1–2 gap years: 2.48 (0.94); >2 gap years: 2.63 (0.68); career changer: 2.25 (0.96)	0.517
Q13: Likelihood of Applying Strategies		
First-generation vs non-first-generation	3.09 (1.11) vs 3.15 (1.23)	0.83
Prior formal study strategy instruction vs none	3.28 (1.25) vs 2.98 (1.04)	0.249
Path to medical school	Direct: 3.27 (1.16); 1-2 gap years: 3.09 (1.21); >2 gap years: 2.95 (0.91); career changer: 3.00 (1.63)	0.463

Greater engagement with the materials was associated with greater perceived impact. Because email engagement was substantially higher than engagement with PDF materials and podcasts and represented the primary delivery modality of the intervention, subsequent correlational analyses focused on email engagement. The number of emails read demonstrated significant positive correlations with perceived helpfulness of the series (r = 0.503, p < 0.001), perceived introduction of new techniques (r = 0.302, p = 0.007), influence on study approach (r = 0.522, p < 0.001), and likelihood of applying strategies in medical school (r = 0.612, p < 0.001). Perceived helpfulness also had significant positive correlations with learning new techniques (r = 0.510), influence on study approach (r = 0.657), and likelihood of application (r = 0.733). These findings are shown in Table 6.

**Table 6:** Correlations between engagement and perceived impact

Engagement measure	r	p-value
Emails read vs. perceived helpfulness	0.503	< 0.001
Emails read vs. new techniques	0.302	0.007
Emails read vs. influence on study approach	0.522	< 0.001
Emails read vs. likelihood of applying strategies	0.612	< 0.001

## Discussion

This study evaluated the feasibility and perceived impact of an eight-week, AI-assisted study strategies curriculum designed for incoming medical students. Students engaged with the materials to varying degrees, and greater engagement was consistently associated with higher perceived usefulness across multiple domains, including exposure to new techniques, influence on study approach, and anticipated application of strategies during medical school. While the present study did not assess objective changes in study behaviors or academic outcomes, these findings suggest that incoming students perceived value in receiving structured instruction on evidence-based learning techniques prior to matriculation. Prior studies of pre-matriculation programs have demonstrated that early academic interventions can improve first-year performance, particularly among students entering with lower traditional academic metrics or from underrepresented backgrounds [4].

A substantial body of learning science research demonstrates that strategies such as spaced practice, retrieval practice, and interleaving improve learning outcomes in health professions education [5]. Systematic reviews across multiple disciplines have shown consistent benefits of these strategies on academic performance, particularly when learners receive explicit instruction on how and when to apply them [6]. Although the present study did not measure objective changes in study behaviors, academic performance, retention, or well-being, students reported exposure to new techniques and an increased likelihood of applying them during medical school. Prior work suggests that structured learning strategy training can improve metacognitive awareness, increase adoption of effective study practices, and decrease reliance on less effective methods such as rereading and highlighting [3, 7].

Notably, perceived impact did not differ by first-generation status, prior formal instruction in study strategies, or path to medical school. These findings suggest that the curriculum was perceived similarly across the demographic and educational subgroups examined. However, the study was not designed or powered to detect subgroup differences, and the absence of statistically significant differences should not be interpreted as evidence of equivalent benefit across populations. Previous studies have demonstrated that learning strategy interventions and pre-matriculation programs may disproportionately benefit academically disadvantaged students and can reduce performance gaps over time [4, 7]. Future research should further explore whether similar interventions have differential effects across learner populations.

The curriculum’s online and multimodal design may have further contributed to its accessibility and perceived utility. Prior evidence indicates that blended and technology-enhanced learning formats are associated with improved knowledge outcomes compared with traditional instruction alone, particularly when interactive and self-directed components are incorporated [8]. However, engagement differed substantially across the curricular modalities. Students reported reading an average of 3.58 emails but reviewing only 2.29 PDFs and listening to 1.03 podcasts. One possible explanation is that emails required the least effort to access and consume, whereas PDFs and podcasts demanded greater time and cognitive investment. Students may also have preferred written content that could be reviewed quickly and revisited as needed. These findings highlight the differing levels of effort required to engage with each modality and suggest that brief, low-friction educational formats may be particularly well suited for pre-matriculation interventions. Future iterations of the curriculum may benefit from prioritizing concise digital content or restructuring audio materials into shorter micro-learning segments.

The use of large language models to assist in curriculum development represents a potentially scalable approach within medical education. In this study, LLMs were used to assist with the development of standardized educational materials rather than to provide individualized instruction. AI-assisted drafting facilitated the translation of learning-science literature into learner-facing content, while educator review and revision ensured accuracy, alignment with the educational literature, and appropriateness for the target audience. This workflow may reduce faculty burden associated with developing educational resources while maintaining human oversight of content quality. As AI tools continue to evolve, future work should examine how these technologies can best support the development and dissemination of evidence-based educational materials. Beyond curriculum development, emerging evidence suggests that LLMs may also support learners directly. For example, LLMs can assist students by generating practice questions, case-based scenarios, mnemonics, and other active learning exercises that promote engagement and knowledge retention [9]. Future research should explore in depth how these tools can be integrated responsibly to support both educators and learners in medical education.

This study has several limitations that should be considered when interpreting the findings. The project was performed at a single institution and used a single-group, cross-sectional survey design, which limits the ability to establish causality or directly attribute perceived benefits to the intervention itself. The absence of a control group and baseline assessment further limits interpretation of the observed outcomes. Because outcomes relied on post-intervention self-report, the results may have been influenced by recall bias, response bias, social desirability bias, and subjective perception. Engagement measures were also self-reported, and objective usage analytics such as email open rates, PDF downloads, or podcast listening metrics were not available, limiting the ability to verify actual engagement with the curricular materials. In addition, perceived benefits may not correspond to measurable changes in study habits, academic outcomes, long-term retention of study strategies, or learner well-being. Student participation and engagement with the curriculum components varied considerably, with particularly low engagement observed for podcast content. Voluntary participation may have further introduced selection bias, as students who were more academically motivated or more concerned about preparedness may have been more likely to participate and provide favorable responses. Finally, the survey instrument was developed specifically for this study, and the AI-assisted educational materials were created through an iterative process rather than standardized prompts, which may reduce reproducibility of the intervention. In addition, multiple subgroup and correlational analyses were conducted without formal correction for multiple comparisons; therefore, these findings should be interpreted as exploratory and hypothesis-generating. Future studies using prospective, controlled, and multi-institutional designs would help better assess the long-term effectiveness and broader applicability of pre-matriculation study strategy curricula.

## Conclusions

The eight-week, AI-assisted study strategies curriculum was feasible to deliver and was perceived as useful by participating students, with greater perceived benefit reported among those who engaged more extensively with the materials. Students reported exposure to new study techniques, perceived changes in their approach to learning, and an increased likelihood of applying evidence-based strategies during medical school. Engagement was highest with email-based content, suggesting that brief, low-friction educational formats may be particularly well suited for pre-matriculation interventions.

Large language models provided a scalable means of assisting with curriculum development while allowing educator oversight of accuracy and educational alignment. These findings suggest that AI-assisted approaches may help support the creation and dissemination of evidence-based educational resources for incoming medical students. Future research should evaluate whether pre-matriculation study strategies curricula lead to measurable changes in study behaviors, academic performance, well-being, and the transition to medical school.

## Appendices

Appendix 1 - study strategies curriculum survey

Welcome to the Study Strategies Curriculum Survey.

This brief survey is part of a quality improvement effort to enhance the email series on learning and study strategies sent to incoming medical students. Your responses are anonymous and will be used in aggregate to improve future content. There are no right or wrong answers - please respond honestly based on your own experience.

Section 1: Background Information

What was your path to medical school?

·      I came directly from undergraduate studies.

·      I took 1-2 gap year(s).

·      I took more than 2 gap years.

·      I am a career changer (previously worked in a different field).

Are you the first in your family to attend medical school?

·      Yes

·      No

Have you received formal instruction on study strategies before this curriculum? ((e.g., in a course, workshop, seminar, or specific program)

·      Yes

·      No

How many of the 8 Study Strategies emails did you read or listen to? (Use the slider below to select a number from 0 to 8) * Emails (0-8)

How many of the 8 Study Strategies PDF summaries did you read or listen to? (Use the slider below to select a number from 0 to 8) * PDF summaries (0-8)

How many of the 8 Study Strategies podcasts did you read or listen to? (Use the slider below to select a number from 0 to 8) * Podcasts (0-8)

Section 2: General Feedback

7.     Which components of the emails did you find most helpful? (Select all that apply)

·      Learning and study strategies

·      Research article summaries

·      Podcast reviews

·      Practical application tips

·      Other (please specify)

8.     How helpful did you find the email series in preparing for your medical studies?

·      Not helpful at all

·      Slightly helpful

·      Moderately helpful

·      Very helpful

·      Extremely helpful

9.     How likely are you to recommend this email series to other incoming medical students?

·      Not at all likely

·      Slightly likely

·      Moderately likely

·      Very likely

·      Extremely likely

Section 3: Content and Impact

10.  Did the series introduce you to new study techniques that you plan to use?

·      No

·      Somewhat

·      Yes

11.  Did the research articles and podcast reviews enhance your understanding of the topics discussed?

·      No

·      Somewhat

·      Yes

12.  To what extent did the information in the series influence or change your approach to studying or learning?

·      Not at all

·      Slightly

·      Moderately

·      Significantly

·      Extremely

13.  How likely are you to apply the strategies discussed in the email series to your studies in medical school?

·      Not at all likely

·      Slightly likely

·      Moderately likely

·      Very likely

·      Extremely likely

14.  How relevant did you find the research articles included in each email?

·      Not at all relevant

·      Slightly relevant

·      Moderately relevant

·      Very relevant

·      Extremely relevant

Section 4: Pacing, Engagement, and Design

15.  How well did the pacing of the emails align with your ability to absorb the information?

·      Not well at all

·      Slightly well

·      Moderately well

·      Very well

·      Extremely well

16.  Did you find the content engaging and easy to understand?

·      Not at all engaging or easy to understand

·      Slightly engaging and easy to understand

·      Moderately engaging and easy to understand

·      Very engaging and easy to understand

·      Extremely engaging and easy to understand

17.  How would you rate the clarity and overall presentation of the emails (layout, formatting, visuals, etc.)?

·      Very poor

·      Poor

·      Average

·      Good

·      Excellent

18.  How would you rate the overall visual design and layout of the emails (readability, formatting, images, spacing)?

·      Very poor

·      Poor

·      Average

·      Good

·      Excellent

19.  How would you describe the tone of the emails?

·      Very relatable and encouraging

·      Clear and professional

·      Neutral

·      Too casual

·      Too formal or impersonal

Section 5: Open ended (Optional)

20.  How could the research articles or podcast reviews be improved to better support your learning as an incoming medical student?

21.  Were there any specific topics, strategies, or elements in the emails that you found unhelpful or confusing? Please explain.

22.  What was your least favorite aspect of the email series, and why?

23.  Did the podcast reviews of the research articles enhance your understanding of the topics? If so, how?

Appendix 2: sample email/PDF content

Good morning future Students,

Happy Study Strategy Saturday! Welcome back to your weekly dose of study tips tailored for you, our incoming medical students. Each Saturday, we’re here to equip you with smart, practical strategies to conquer your coursework and excel in med school. Let’s dive into today’s tip and keep your study game strong!

Practical Learning Strategies for Medical School

Medical school requires mastering extensive factual knowledge while ensuring long-term retention for clinical practice. Effective learning strategies emphasize active engagement, structured repetition, and practical application. Below are three evidence-based techniques to maximize learning and retention, along with multimedia resources for further exploration.

1. Spaced Repetition

Spaced repetition leverages the forgetting curve by reviewing material at increasing intervals to reinforce memory. This technique is ideal for retaining the high volume of factual knowledge in medical school, such as anatomy or pharmacology.

Putting it into practice:

Use tools like Anki to create digital flashcards with questions on one side and answers on the other (e.g., "What is the mechanism of action of metformin?").

Schedule reviews based on the software’s algorithm, prioritizing daily consistency (e.g., 20-30 minutes per day).

Focus on high-yield topics and integrate new cards gradually to avoid overwhelming yourself.

Check Out: Anki

Med School Insiders "Anki Tutorials" Playlist covers how to set up Anki for medical school and optimize card creation.

2. Active Recall

Active recall involves retrieving information from memory without cues, strengthening neural pathways and improving retention. This contrasts with passive review (e.g., rereading notes) and is highly effective for mastering complex medical concepts.

Putting it into practice:

During study sessions, close your notes and attempt to explain or write key concepts from memory (e.g., the steps of the Krebs cycle).

Use practice questions from resources like AMBOSS and UWorld to simulate exam conditions and identify weak areas.

After each session, review incorrect answers to reinforce learning and adjust your study focus.

Check Out:  How to do ACTIVE RECALL Effectively? (4 Techniques worked for me)

3. Self-Testing

Self-testing involves regularly assessing your knowledge through quizzes, practice exams, or problem sets to reinforce learning and identify gaps. This technique enhances retention by simulating high-stakes environments and promoting active engagement with material.

Putting it into practice:

Create or use question banks (e.g., Kaplan, Lecturio..Anki, Amboss!) to test yourself on topics like physiology or pathology after studying.

Set timed practice sessions to mimic exam conditions, aiming for 50-100 questions per session.

Review explanations for both correct and incorrect answers to deepen understanding and adjust study priorities.

General Tips for Success

Prioritize Consistency: Dedicate specific times daily for each technique to build habits.

Integrate Techniques: Combine spaced repetition with self-testing by using flashcards for quick quizzes, and apply active recall to explain concepts before testing.

Monitor Progress: Track your performance on practice questions and adjust study plans to focus on weaker areas.

Check Out:  3 tips on how to study effectively Ted-Ed

Listen up: Effective Learning Strategies in Medical School 1.4.wav

Read Up: How to Learn in Med School.pdf

These strategies are grounded in cognitive science and widely endorsed by medical education experts. For best results, tailor them to your learning style and maintain a balanced schedule to prevent burnout. Explore the recommended resources to deepen your understanding and refine your study habits.

👀 Check Out: 3 tips on how to study effectively Ted-Ed 

🎧 Listen up: Effective Learning Strategies in Med School

📖 Read Up:  How to Learn in Med School .pdf   

Appendix 3 - sample podcast transcript

Hey everyone and welcome back to the Study Pros Podcast. Today we're going to be talking about something that I think we've all struggled with. How to learn effectively. Especially when it feels like we're just drowning in information. We've got this super interesting article from the Yale Journal of Biology and Medicine. And while it's written, for medical students, I think the insights are really valuable for anyone who has ever... felt overwhelmed by learning for sure which let's be honest it's probably all of us at some point absolutely that feeling of information overload is definitely universal it doesn't matter if you're in school or trying to like level up your skills at work or just someone who loves to learn new things figuring out how to learn smart yeah is a game changer okay so let's break this down the article starts off by talking about how medical students often struggle with those massive volume yeah facts they need to cram into their brains yeah but then it gets really interesting because they make this distinction yeah between factual knowledge yeah and procedural knowledge and that distinction is key think of it this way factual knowledge is like knowing the parts of a car, the names, the functions, it's the what. Procedural knowledge is knowing how to actually drive the car. It's the how and the why. Okay, I'm with you. So the article's point is that while we often learn procedural stuff through hands-on practice, factual knowledge kind of gets stuck with these less effective methods like reading and highlighting. Which brings us to the dreaded forgetting curve, something we've probably all experienced firsthand. Oh, tell me about it. Herman Ebbinghaus. way back in the day figured out that we forget newly learned information so fast unless we actively fight to keep it right in a halkboard and somebody just comes and wipes it clean that's a great analogy and without practice that knowledge truly does fade quickly but here's the good news okay memory is like a muscle the more we use it the right way the stronger it gets the article even shares this wild example of a student who through like crazy dedicated practice yeah could remember a sequence of 80 random digits. It's like, what? That's a feat of memory. And it segues perfectly into one of the most powerful strategies for learning, the testing effect. Okay, I'm intrigued. Testing is more effective than studying. How does that work? Well, studying can give a false sense of knowing. We read something, we highlight it, and we think, got it. But testing forces your brain to retrieve the information. It's active, not passive. That active recall process strengthens the pathways to that knowledge. So it's not just about stuffing the information in, it's about making sure we can pull it back out when we need it. Precisely. The article highlights a study. Where one group just studied a list of words four times while another group studied it once and then took three tests on it. And the results, night and day. The testing group's recall was way higher. Wow. That's amazing. So even if we're not getting everything right on those tests, the act of trying to remember is actually making the learning stick better. Exactly. It's not about being perfect. It's about engaging with the material. Even unsuccessful recall attempts, as long as you eventually see the correct answer, helps solidify the learning. So don't be afraid to test yourself, even if you think you're going to mess up as part of the process. This leads us to our next strategy, right? Yes. Active recall. Active recall is all about being proactive with your learning. Instead of passively reading or reviewing, you're actively challenging yourself to pull information from your memory. I'm starting to see a pattern here. It's all about making our brains work. You got it. The harder your brain has to work to retrieve something, the stronger the memory becomes. It's like forging a mental pathway. The more you tread that path, the clearer and stronger it gets. The article gave this example. of learning the side effects of beta blockers, which are medications for heart conditions. They said writing those side effects down from memory is way more effective than just reading them over and over again. That makes sense, right? Just reading is like glancing at a map, but writing from memory is like navigating the actual terrain. It forces you to recall the details and solidify the connections. They even mentioned this study where people tried to learn names at a party. That's my worst nightmare. But in the study, the people who actively tried to recall the names throughout the party were way better at remembering them later on. So engaging actively with information leads to better retention. That's a principle that applies to all kinds of learning. Okay, so testing ourselves. Yeah. And actively recalling information. I got it. What's the third strategy? Space repetition. It's about fighting that forgetting curve head on instead of cramming. You space out your study sessions over time. So no more all-nighters before exams, huh? Well, ideally not. They compared steady versus expanding retrieval practice. Steady means reviewing at consistent intervals, like every day. Expanding means gradually increasing the time between sessions, maybe reviewing today, then two days later. than a week later. And which one was more effective? You guessed it. Expanding retrieval. Giving yourself that space allows some forgetting to happen, which actually forces your brain to work harder when you do review. And that effort makes the memory stick better. So it's about embracing that, forgetting, knowing that it's actually helping us learn in the long run. Precisely. It's counterintuitive. That's how our brains work. And they pointed out that this is especially helpful for information that's easily confused with other stuff. Like when you're studying multiple subjects with similar concepts. Exactly. Space repetition helps those individual pieces of knowledge really solidify and not get jumbled up. in your memory so to recap we've got testing active recall and spaced repetition three powerful tools for learning effectively and it's not just for academics these strategies can be applied to learning anything a new language a musical instrument literally anything it's like having a mental toolkit for learning and the best part it gives you agency you're not just passively absorbing information right you're actively shaping how your brain learns and remembers i love that taking control of our own learning so to kind of wrap up this part of our discussion on effective learning. I want to leave our listeners with a question. Okay. How can you incorporate these strategies, testing.active recall and spaced repetition into your learning routine? That's a great question to reflect on because whether you're a student, a professional, or just someone who loves to learn, these techniques can make a real difference in how effectively you acquire and retain knowledge. Stay tuned, everyone. We'll be back after a short break to continue our exploration of afflictive learning strategies. Welcome back to the Study Pros podcast. Before the break, we were talking about these three awesome learning strategies, testing, active recall, and spaced repetition. And, you know, it got me thinking about... my own experience is trying to cram for exams back in the day. Yeah. Like I wish I had known about these techniques then. I hear you. It's amazing how much of a difference the right strategies can make. It's not just about how much time you spend studying. It's about how you study. Absolutely. Speaking of which, one of the studies mentioned in the article really stuck with me. Okay. It involved people learning about different geographical regions. And what's fascinating was that the benefits of retrieval practice that we talked about earlier were even stronger when the information was vulnerable to getting mixed up with other similar information. Now, that's interesting. What do you mean by vulnerable to getting mixed up? Well, imagine you're trying to learn all about Antarctica, the climate, the wildlife, the geography. But then you immediately dive into learning about other similar regions like Greenland or even the Arctic Circle. All those facts about ice and penguins and polar bears can start to blur together in your mind. Ah, I see. So you're saying that space retrieval is especially helpful when you're dealing with lots of related but distinct pieces of information. Exactly. Because it helps you solidify. those individual pieces of knowledge and prevent them from becoming one big jumbled mess in your memory. That makes a lot of sense. It's like creating clear and distinct mental file folders for each piece of information rather than just dumping everything into one giant overflowing folder. I love that analogy. And one thing that surprised me in the article was that even though all this research supports these effective learning strategies, they're often not taught in medical school. You're kidding. You'd think that of all places, medical school would be at the forefront of teaching students how to learn effectively given the sheer amount of information they need to absorb. I mean, wouldn't it make sense to give these future doctors the best possible tools for learning and retaining all that critical knowledge. It certainly seems like a missed opportunity. I wonder why these evidence -based techniques haven't been more widely integrated into medical education. The article suggests it might be due to time constraints. Okay. You know, medical school curricula are already jam-packed. Right. And it can be tough to justify adding more content, even if it's about something. As fundamental as learning how to learn. I understand that perspective. But I'd argue that teaching students how to learn is just as important, if not more so, than teaching them specific facts. After all, medical knowledge. is constantly evolving. What doesn't change is the ability to learn and adapt. So true. It's like the old saying, give a man a fish and you feed him for a day. Teach a man to fish and you feed him for a lifetime. Teaching effective learning strategies is like giving students the tools to become lifelong learners, quick to navigate any intellectual challenge that comes their way. Absolutely. And think about the implications beyond just academic performance. Knowing how to learn effectively can empower people in all areas of life, from adapting to new job requirements to picking up new hobbies and interests. It's like a superpower that can benefit us. In countless ways. The article also mentioned that, ideally, a program teaching these learning strategies should be implemented early on in medical school. That makes perfect sense. Why wait until students are already struggling to keep up with the demands of their studies? Giving them these tools from the start could help them develop good learning habits early on and set them up for success. And they even proposed that the program itself should be taught using the same principles of active learning testing and spaced repetition. meta, using the very techniques they're teaching to deliver the instruction. What a brilliant way to demonstrate the power of these methods firsthand. A living, breathing example of how effective these techniques can be. They even suggest that the program could be adapted to different stages of medical education, focusing on specific challenges and skills needed at each stage. That's a really thoughtful approach. It recognizes that learning is a dynamic process. And the strategies we use need to evolve as we progress through different levels of complexity. What I really appreciate is how they emphasize the importance of creating a supportive learning environment and raising awareness. About the learning process itself. It's not just about memorizing facts. It's about understanding how our brains learn best. Right. And then using that knowledge to optimize our learning experience. So shifting from a mindset of passive absorption to one of active engagement and ownership. And that's a powerful shift. And that can benefit anyone, regardless of their field of study or profession. I love that taking ownership of our learning. It's empowering. And, you know, the article wraps up by acknowledging that these learning strategies are just a starting point. There's a lot more research being done. In the field of cognitive neuroscience and education. I, for one, am excited to see what new discoveries emerge and how they can further enhance our understanding of learning. It's fascinating to think about how we can leverage our knowledge of the brain to create even more effective learning environments and strategies. And it's a field with immense potential to impact not just students, but anyone who's eager to learn and grow throughout their lives. That's exciting. Imagine a world where everyone has the knowledge and tools to learn effectively. What a world that would be. But even without that perfect world, we have the power. To take charge of our own learning journeys right now. That's such an important message. We don't have to wait for the education system to catch up. We can start applying these evidence-based techniques today in any area of our lives. We can test ourselves, practice active recall, space out our study sessions, and really engage with the material we're learning. It's about becoming active participants in our own learning, not just passive recipients of information. And yeah, that brings us to the heart of what we've been talking about today. We've explored these incredible strategies for learning more effectively, testing active recall and spaced repetition. And now, the question for our listeners is, how are you going to put these strategies into action? What changes can you make to your own learning routine starting today to make your learning more efficient, enjoyable, and ultimately more meaningful? That's something to ponder. We'll be right back to wrap things up on the Study Pros podcast. Welcome back to the Study Pros podcast. I'm so energized after that discussion about how we can actually hack. our own learning process. It's like we've been handed the secret code to unlock our brain's full potential. I agree. It is incredibly empowering to realize that we're not just stuck with the learning habits we've always had. We can actively reshape them using these evidence-based strategies. Exactly. So before we wrap up, I think, a quick recap of the three learning powerhouses we decussed would be super helpful for our listeners. That sounds good to me. Let's give everyone a toolkit they can walk away with and start using today. Strategy number one, the testing effect. We talked about how testing yourself, instead of just passively rereading or highlighting, forces your brain to actively retrieve information. And that active retrieval is like a mental workout. It strengthens those neural connections and it makes the learning stick. It's like the difference between looking at a photo of a place you've been and actually retracing your steps on a map. That active effort of recalling the route makes the memory much more vivid and lasting. Perfect analogy. And remember, even if you don't, ace those self-tests right away. The effort itself is beneficial. Mistakes are just stepping stones on the path to mastery. Absolutely. Embrace those stumbles. They're proof that your brain is working hard. Now, for strategy number two, we have active recall. It's all about challenging yourself. to pull information from your memory without relying on your notes or textbooks. It's like putting your brain through a boot camp, right? You got it. No crutches, just pure mental muscle. And the harder you have to work to recall something, the deeper it gets etched into your memory. So ditch the passive highlighting and put your brain to the test. Try explaining concepts out loud, teaching them to an imaginary student, or writing down everything you remember without peeking. So many great ideas. And finally, I have strategy number three, spaced repetition. This one is all about outsmarting the forgetting curve by spacing out. your study sessions over time instead of cramming everything in it was and as we discussed yeah gradually increasing the time between those sessions that's expanding retrieval practice seems to be the most effective approach it gives your brain just enough time to forget a bit so when you do come back and the material it's like fresh challenge and that challenge forces your brain to work even harder leading to even stronger memories it's like a mental game of hide and seek exactly the information might try to hide But your brain will always find it. And remember, spaced repetition is especially powerful when you're dealing with lots of information that's similar or easily confused. It helps to create clear, distinct mental compartments for each concept so they don't all blend together. So there you have it, folks. Our three learning superheroes. Chesting, active recall, and spaced repetition. It's like having a brain-boosting trio on your side. And the most exciting part is that these strategies are not just for students or academics. They're for anyone who wants to learn anything. A new language, a new skill, a new recipe. the principles apply across the board. It's about harnessing the incredible power of our brains and becoming active architects of our own learning experiences. And with that thought, I think we've reached the end of our journey for today. It's been an absolute pleasure exploring these fascinating learning strategies with you. The pleasure was all mine. And to our amazing listeners, we hope you feel inspired to put these strategies into practice and unlock your own learning superpowers. Thanks for joining us on the Study Pros Podcast. Until next time, happy learning.
